# Season of birth is associated with multiple sclerosis and disease severity

**DOI:** 10.1177/20552173211065730

**Published:** 2021-12-22

**Authors:** P Stridh, J Huang, AK Hedström, L Alfredsson, T Olsson, J Hillert, A Manouchehrinia, I Kockum

**Affiliations:** Center of Molecular Medicine, 97092 Karolinska University Hospital, Solna, Sweden; Department of Clinical Neuroscience, 27106 Karolinska Institutet, Stockholm, Sweden; Department of Clinical Neuroscience, 27106 Karolinska Institutet, Stockholm, Sweden; Institute of Environmental Medicine, 97092 Karolinska Institutet, Stockholm, Sweden; Center of Molecular Medicine, 97092 Karolinska University Hospital, Solna, Sweden; Department of Clinical Neuroscience, 27106 Karolinska Institutet, Stockholm, Sweden

**Keywords:** multiple sclerosis, month of birth, season, severity, vitamin d, birth cohort

## Abstract

**Background:**

The latitude gradient in multiple sclerosis incidence indicates that low sun exposure and therefore vitamin D deficiency is associated with multiple sclerosis risk.

**Objective:**

Investigation of the effect of month of birth, which influences postnatal vitamin D levels, on multiple sclerosis risk and severity in Sweden.

**Methods:**

Patients and population-based controls were included from three nationwide cohorts. Differences in month of birth between cases and controls were analyzed using logistic regression and examined for effect modification by calendar year and geographic region at birth.

**Results:**

Males had a reduced risk of multiple sclerosis if born in the winter and increased risk if born in the early fall. Individuals born before 1960 had an increased risk if born in summer or fall. Being born in late summer and early fall was associated with more severe disease.

**Conclusions:**

We identified a birth cohort effect on the association between the month of birth and multiple sclerosis, with a more significant effects for births before 1960. This coincides with a period of lower breastfeeding rates, recommended intake of vitamin D, and sun exposure, resulting in a lower vitamin D exposure during the fall/winter season for infants born in the summer.

## Introduction

Vitamin D modulates several immune processes with deficiencies leading to impaired immune responses against infections and increased risk of certain autoimmune disorders.^1^ Vitamin D deficiency is also associated with an increased risk of multiple sclerosis (MS), a chronic inflammatory disease resulting in central nervous system (CNS) demyelination. MS patients often have lower levels of circulating vitamin D, including the stabler hydroxylated form, 25(OH)D, and the active form, 1,25(OH)_2_D.^1^ The higher frequency of genetic predisposition to low vitamin D expression among MS patients^2–4^ and the latitude gradient in MS prevalence with increased risk at higher latitudes and lower ultraviolet radiation levels further supports the pathological association with vitamin D, which is primarily produced photochemically.^5^

The extent of vitamin D involvement in MS pathogenesis remains unclear. However, sunlight exposure, particularly during adolescence or early life, has been implicated in MS risk.^6,7^ This is further evidenced by the increased risk among offspring of mothers with low vitamin D intake during pregnancy, suggesting the early involvement of vitamin D during prenatal development of the immune system.^6^ Previous studies have observed an association between the season or month of birth and the risk of developing MS, possibly due to differences in sun exposure during pregnancy.^8–11^ However, recent studies have not been able to replicate such findings.^12–14^ In contrast to risk, the long-term implications of vitamin D and sun exposure on MS disease severity and treatment response are inconsistent.^15^ This study examines the association between the month of birth and the risk of MS development and disease severity in Sweden.

## Materials and methods

### Cohort description

MS patients were included from three Swedish cohorts: Genes and Environment in MS (GEMS) is a national prevalence-based study of MS patients recruited from the Swedish MS registry between November 2009 and November 2011;^3^ Epidemiologic Investigation in MS (EIMS) is an incidence-based study enrolling newly diagnosed MS patients from 42 neurological clinics across Sweden;^3^ and the Immunomodulation and MS Epidemiology (IMSE) study follows patients on immunomodulatory treatments for MS to examine clinical, genetic, and environmental factors that influence treatment response.^16^ Population-based controls were matched to cases based on age (± 5 year intervals), sex, and residence area at the time of inclusion. Descriptive characteristics for each cohort are provided in **Table 1**.

**Table 1.** Description of cohort

	**GEMS**		**EIMS**		**IMSE**	
	**MS cases**	**Controls**	**MS cases**	**Controls**	**MS cases**	**Controls**
N	7893	8708	3379	8505	2209	3882
Female	5673 (72%)	6257 (72%)	2375 (70%)	5825 (68%)	1518 (69%)	2699 (70%)
*Disease characteristics and severity*						
Age (onset)	32.6 ± 10.6	-	33.6 ± 10.6	-	31.6 ± 10.5	-
Duration	13.6 ± 11.0		4.5 ± 6.2		4.7 ± 5.3	
EDSS, ≥3	3777 (54%)	-	512 (23%)	-	127 (25%)	-
EDSS, ≥6	1788 (26%)	-	59 (3%)	-	18 (4%)	-
MSSS, ≥3	3940 (58%)	-	1059 (48%)	-	265 (55%)	-
MSSS, ≥6	2020 (30%)	-	462 (21%)	-	116 (24%)	-
ARMSS, ≥3	4716 (67%)	-	1182 (54%)	-	339 (67%)	-
ARMSS, ≥6	2544 (36%)	-	396 (18%)	-	145 (29%)	-
*Season of Birth*						
Spring	2129 (27%)	2465 (28%)	972 (29%)	2417 (28%)	645 (29%)	1106 (28%)
Summer	2039 (26%)	2151 (25%)	804 (24%)	2082 (24%)	542 (25%)	950 (24%)
Fall	1879 (24%)	1988 (23%)	774 (23%)	1928 (23%)	519 (23%)	901 (23%)
Winter	1846 (23%)	2104 (24%)	829 (25%)	2078 (24%)	503 (23%)	925 (24%)

Count with percent frequency or mean with standard deviation are provided for each multiple sclerosis (MS) cohort: GEMS, Genes and Environment in MS; EIMS, Epidemiologic investigation in MS; and IMSE, Immunomodulation and MS Epidemiology. Expanded disability status scale (EDSS), MS severity score (MSSS), and age-related MS severity (ARMSS) score were dichotomized by ≥3 and ≥6.

Data on disease characteristics were extracted from the Swedish MS registry, including age at onset, disease course, disease duration, and disability level as measured by the first available expanded disability status scale (EDSS) score. Disease severity was characterized using both the MS severity score (MSSS) and the age-related MS severity (ARMSS) score. Year, month, and region of birth were determined through their government-issued personal identification number.

### Ethical approvals and patient consents

The Stockholm Regional Ethical Review Board has approved all studies under ethical permits Dnr 2017/1349-32 (EIMS, 2017-06-28), Dnr 2017/1350-32 (GEMS, 2017-06-28), and Dnr2017/1426-32 (IMSE, 2017-07-06)), and study participants have provided informed and written consent.

### Data availability

Anonymized data used in this study will be shared upon request to the corresponding author by any qualified investigator pending Institutional Review Board approval.

### Statistical analysis

Differences in the distribution of month of birth between MS cases and controls were analyzed using a logistic regression model. The season of birth was defined by three-month intervals, including winter (Dec/Jan/Feb), spring (Mar/Apr/May), summer (Jun/Jul/Aug), and fall (Sep/Oct/Nov). Analyses were stratified by calendar year and the geographic region at birth. Three regions were defined based on a latitude gradient: north (Norrland, range 60.4-69.1 N), central (Svealand, range 58.7–62.2 N), and south (Götaland, range 55.4–59.2 N). Two cut-offs, ≥3 and ≥6, were used to dichotomize disability and severity measures. Association between the month of birth and age at disease onset among MS cases were assessed using a linear regression model. All statistical analyses were performed using R v.4.0.2 (R Core Team. Vienna, Austria).

## Results

In an overall analysis with 13,481 MS cases and 21,095 matched controls, month or season of birth was not significantly associated with risk of MS (**Table 2**). A suggestive protective association was observed among those born during the early winter (Nov/Dec/Jan: OR = 0.96, CI_95%_ = 0.91–1.01, P = 0.09), mainly January (OR = 0.93, CI_95%_ = 0.86–1.01, P = 0.07). However, sex-stratified analyses showed that in males MS risk was lower for those born in the early winter (Nov/Dec/Jan: OR = 0.90, CI_95%_ = 0.82–0.99, P = 0.03) and March (OR = 0.87, CI_95%_ = 0.75–1.00, P = 0.04) while increased for early fall (Aug/Sep/Oct: OR = 1.12, CI_95%_ = 1.02–1.23, P = 0.01; October: OR = 1.17, CI_95%_ = 1.01–1.36, P = 0.03). No such association was observed for females (P > 0.15). All analyses were also corrected for sex and cohort; however, no significant differences were observed.

**Table 2.** Month of birth and risk of MS among the Swedish population.

	MS Cases	Controls	1 Month		3 Months	
Month	n (%)	n (%)	OR	95% CI	OR	95% CI
January	1077 (8.8%)	1802 (8.5)	0.93	(0.86,1.01)	0.97	(0.92,1.02)
February	1138 (8.4%)	1711 (8.1)	1.04	(0.97,1.13)	1.00	(0.95,1.05)
March	1248 (9.3%)	2042 (9.7)	0.95	(0.88,1.03)	0.97	(0.93,1.02)
April	1272 (9.4%)	1970 (9.3)	1.01	(0.94,1.09)	1.02	(0.97,1.07)
May	1226 (9.1%)	1976 (9.4)	0.97	(0.90,1.04)	1.00	(0.95,1.05)
June	1169 (8.7%)	1726 (8.2)	1.07	(0.99,1.15)	1.03	(0.98,1.08)
July	1128 (8.4%)	1798 (8.5)	0.98	(0.91,1.06)	1.02	(0.97,1.07)
August	1088 (8.1%)	1659 (7.9)	1.03	(0.95,1.11)	1.04	(0.99,1.10)
September	1137 (8.4%)	1716 (8.1)	1.04	(0.96,1.12)	1.04	(0.99,1.09)
October	1034 (7.7%)	1571 (7.4)	1.03	(0.95,1.12)	1.00	(0.95,1.05)
November	1001 (7.4%)	1530 (7.3)	1.03	(0.94,1.11)	0.96	(0.91,1.01)
December	963 (7.1%)	1594 (7.6%)	0.94	(0.87,1.02)	0.97	(0.92,1.02)

Odds ratios (OR) and 95% confidence intervals (CI) for the risk of MS by month of birth are determine using a logistic regression analysis. Analysis were performed by one month or three months on a sliding window (identified month plus the two following months).

**Figure 1.**
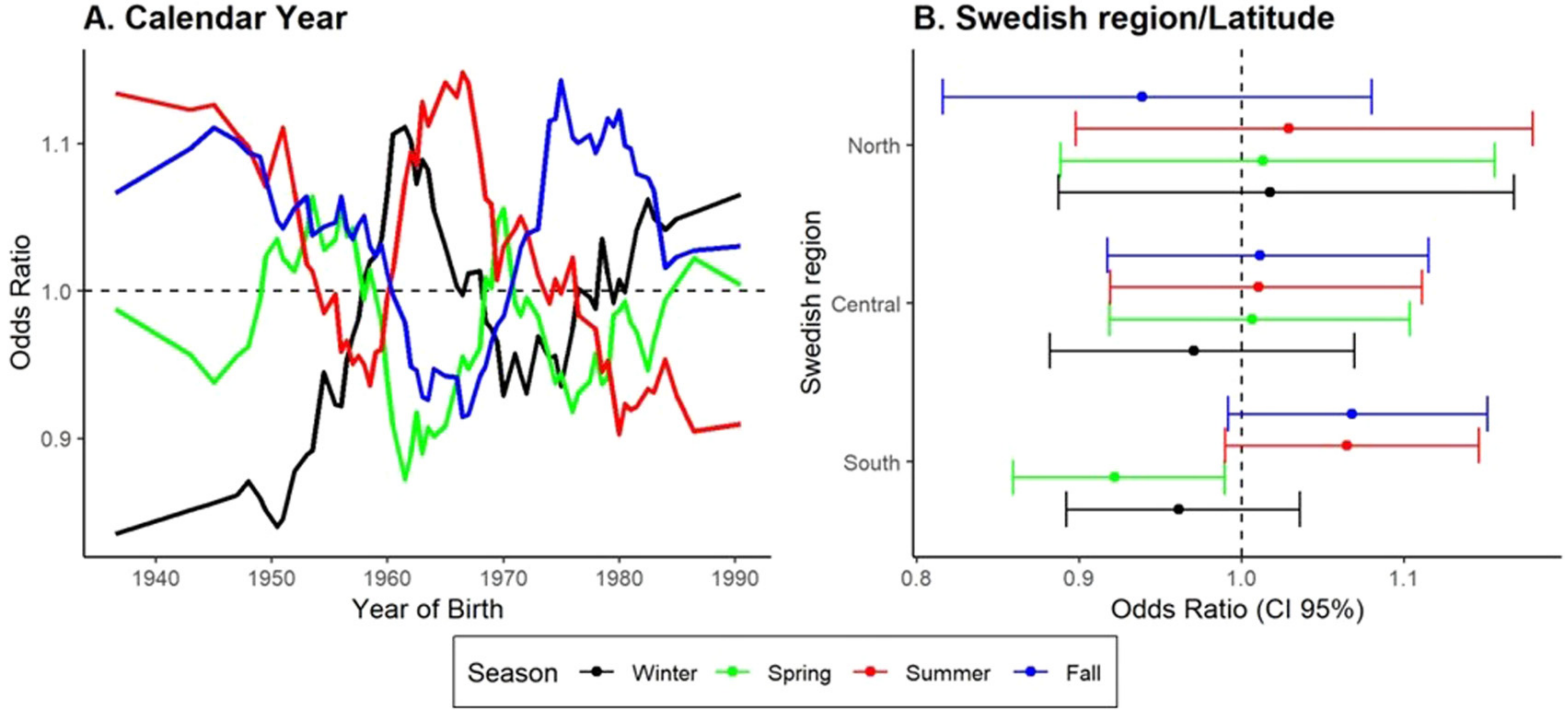
Effects modification of MS risk and season of birth by calendar year and geographic region at time of birth

To examine effect modification by calendar year, overlapping strata based on year of birth were used to investigate the association between season of birth and MS (**Figure 1**). Results suggest a trend with individuals born before 1960 having a decreased risk for MS when born in winter (Dec/Jan/Feb; OR = 0.87, CI_95%_ = 0.79–0.94, P = 0.001) and increased risk when born in fall (Sep/Oct/Nov; OR = 1.14, CI_95%_ = 1.04–1.24, P = 0.003). Both remained significant after multiple testing corrections (false discovery rate correction, P_FDR_<0.05). No significant association was observed among individuals born after 1960 (P > 0.15). When stratified by geographic residence at birth, the association between the season of birth and MS risk was more prominent among those born in southern Sweden (**Figure 1**).

A. Odds ratio for the risk of MS by season of birth (three month intervals) are illustrated stratified by calendar year (20 percentile strata with one percentile steps). B. Odds ratio for risk of MS by season of birth and geographic region (north, central, and south).

**Figure 2.**
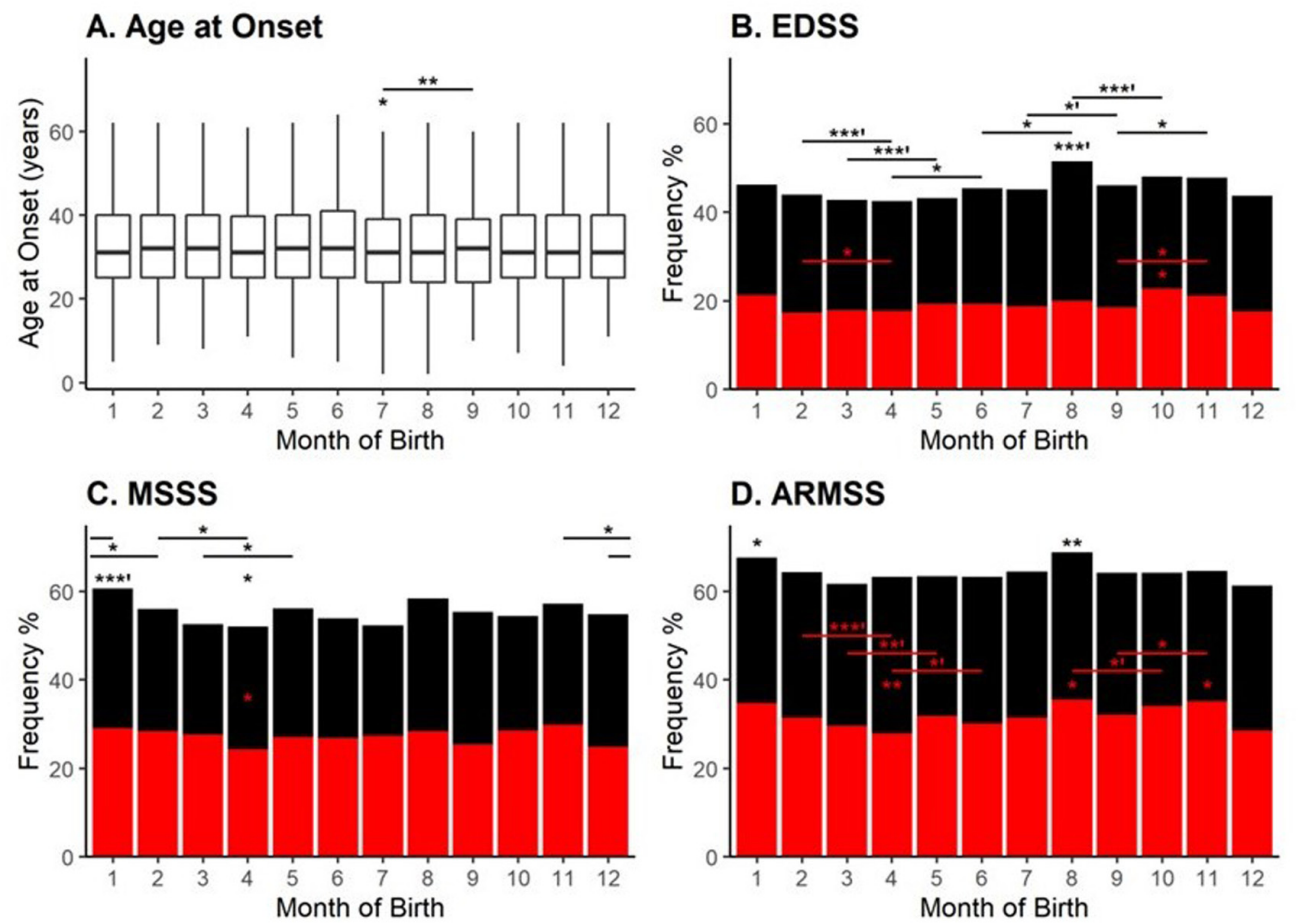
Effects of the month of birth on the age at onset, disease disability, and severity among MS cases

Being born in the summer was associated with a younger age at onset (Jun/Jul/Aug; β = -0.57, P = 0.008) with the peak association in July (β = -0.87, P = 0.01, **Figure 2**). Regarding disease-associated disability and severity, individuals born during late-summer to early-fall (Jul/Aug/Sep/Oct), particularly in August, had accumulated greater disability during the early stages of the disease (P_FDR_ = 0.004) while those born in the spring (Mar/Apr/May) had a lower disability (P_FDR_ = 0.008). Similarly, the probability of an ARMSS score greater or equal to six was also lower among individuals born in spring between February to June while higher among those born in early fall (Aug/Sep/Oct, P_FDR_<0.05). MSSS was only positively associated with those born in January (P_FDR_ = 0.03).

boxplots (panel A) illustrate the distribution of age at onset among MS cases stratified by month of birth. Panels B-C illustrate proportion of MS cases with a disability or severity score ≥3 (black) or ≥6 (red) for EDSS, MSSS and ARMSS respectively. Significance levels correspond to * = 0.05, ** = 0.01, and *** = 0.005 with those P_FDR_<0.05 labelled with (‘).

## Discussion

Our findings indicate that month of birth was associated with MS development primarily among men with a higher risk if born in the early fall and lower risk in the early winter. This association was modified by the calendar year of birth, with both males and females having an increased risk of developing MS if born in the summer or fall before 1960. Differences in the risk by sex may indicate a synergistic interaction between the early environmental exposures associated with the month of birth and sex-based differences in immune development and tolerance,^17^ although the factors and mechanisms involved require further investigation.

The prenatal period is particularly sensitive to vitamin D exposure, since both low dietary intake of vitamin D during pregnancy and low vitamin D levels as a newborn are risk factors for the later development of MS.^6,7,18^ However, it is difficult to distinguish whether the effects of sun exposure, as predicted by the month of birth, occur during pregnancy or after birth as levels are inversely correlated. Low neonatal vitamin D levels measured in a subset of the same cohort as this study were not associated with an increased risk of developing MS later in life, although concerns regarding sample quality have been raised.^19^ This suggests that the effects may not be related to exposure during pregnancy but during the first months after birth.

**Figure 3.**
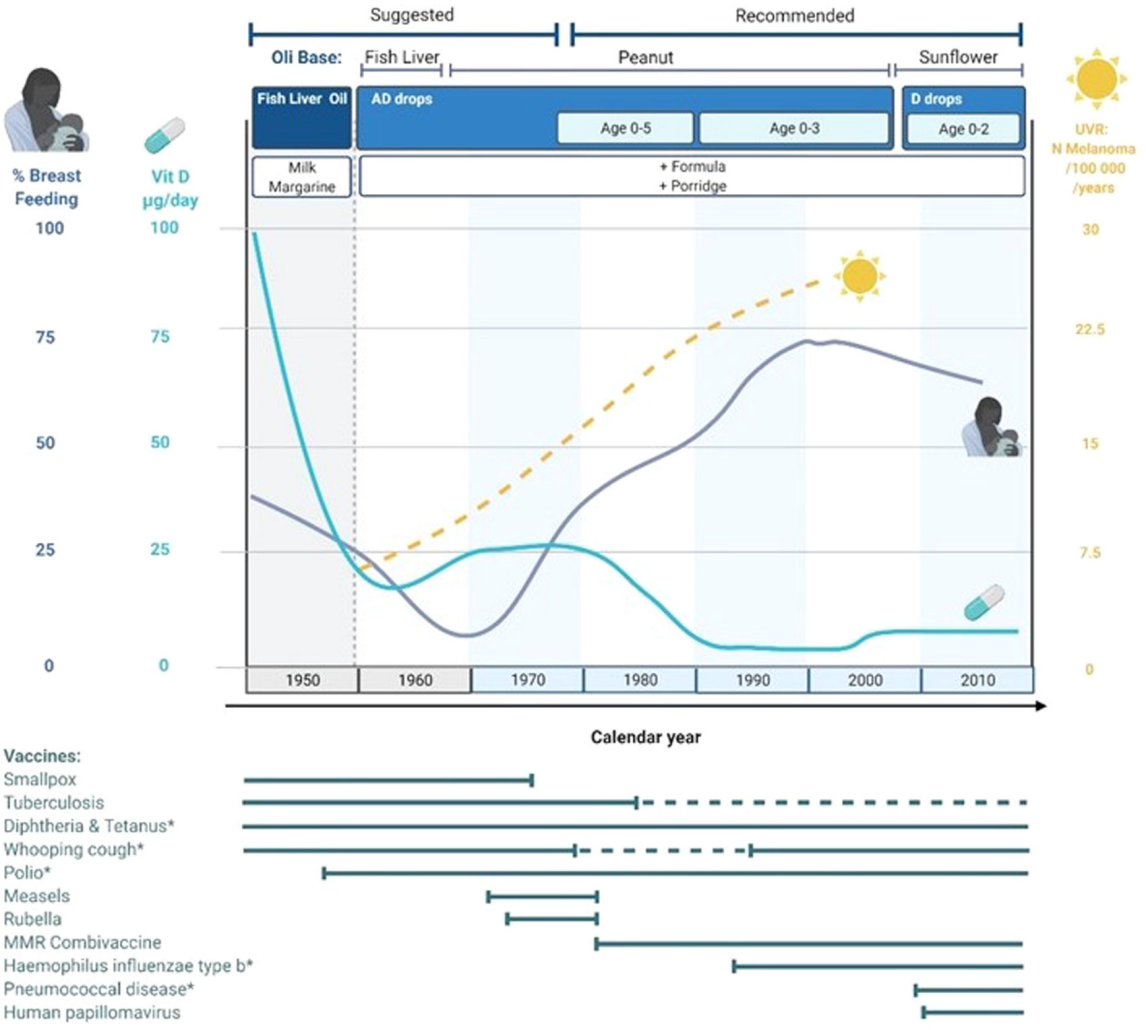
Changes in factors that affect vitamin D levels during the first 6 months of life and immunity to infections, based on data and general recommendations from Sweden years 1950–2019.

Individuals born in the fall have less sunlight exposure during their first six months due to fewer daylight hours and more frequent indoor activity. In our study, these individuals had an increased risk of MS that declined during the 1960s, coinciding with a period of low breastfeeding rate in Sweden (**Figure 3**). At the time, vitamin D fortified infant formula was introduced to prevent rickets in children resulting from vitamin D deficiency. We hypothesize that formula-fed infants are less susceptible to seasonal variability of vitamin D levels in the mother during breastfeeding. Indeed, several studies have reported infants of vitamin D-deficient mothers who are exclusively breastfed are more likely to be vitamin D deficient than formula-fed infants.^20,21^ This is further evidenced by the increased MS risk in fall-born children after the first half of the 1970s, when breastfeeding rates increased again in Sweden from 5% to 38% (**Figure 3**). Although vitamin D supplementation has been available since 1950, the adherence increased after 1978 when the Swedish authorities recommended and distributed daily supplementation for children younger than five years of age.

The x-axis shows time divided in decades and the y-axis is variable specific. Line graph (blue) illustrate the percentage of children that were breastfed at 6 months of age.^31–34^ Line graph (turquoise) illustrate the recommended daily dose (µg/day) of vitamin D supplementation for infants 2 weeks to 1 year of age.^35^ White boxes above the graph indicate Vitamin D-fortified foods and when changes in fortified foods were introduced. Blue boxes above the graph indicate the sources of Vitamin D supplementation for children and the ages during which supplementation was given. Blue bars indicate the oil base used for supplementation (narrow) and whether Swedish authorities suggested or recommended and supplied vitamin D drops for supplementation. Line graph (yellow) illustrate the age standardized incidence of cutaneous melanoma in Sweden in those already diagnosed with a primary cutaneous melanoma in the same decade, as an indicator of high UVR exposure.^23^ Green bars below the graph illustrates vaccinations administered through the public health system (https://www.folkhalsomyndigheten.se/the-public-health-agency-of-Sweden/communicable-disease-control/vaccinations/previous-Swedish-vaccination-programmes/). Vaccines administered before 6 months of age are indicated with *. The adherence is generally high, with >97% vaccinated against the infections included in the child vaccination program (https://www.folkhalsomyndigheten.se/datavisualisering/). The figure was created with BioRender.com.

Active sunbathing and travelling to sunnier climates have also steadily increased since the 1960s. Incidence of cutaneous melanoma, a population-level indicator of high ultraviolet radiation (UVR) exposure, has gradually increased by approximately 5% annually.^22,23^ The rate of subsequent melanomas among patients previously diagnosed with a primary cutaneous melanoma has also steadily increased since the 1960s,^(^^24^ **Figure 3**). Thus, the decrease in MS risk associated with the season of birth after 1960 may reflect added resilience of mothers to seasonal vitamin D deficiency along with the increased use of formula and its fortification with vitamin D . The trend in seasonal differences have also steadily declined from 1975, indicating that the responsible risk factor is changing over time.

Breastfeeding may also have a direct role in the risk of MS.^25^ In a recent study from the same cohort, prolonged breastfeeding was associated with reduced MS risk only among men.^26^ Possible explanations include effects on microbiota known to influence autoimmunity,^27^ Th1 shift in formula-fed children,^28^ and potential molecular mimicry between myelin oligodendrocyte glycoprotein, a MS autoantigen, and bovine butyrophilin in formula.^29^ Differences in the risk association to breastfeeding by sex may indicate developmental differences in the immune system in utero or during childhood^30^ or influence from other potential environmental confounders.^26^ However, the seasonal timing of breastfeeding was not considered in the reported study,^26^ and it is unknown if this effect changes over time.

Associations between MS and month of birth may also be independent of vitamin D. Exposure to UV radiation can also have direct immune-suppressive effects by altering inflammatory cytokine profiles and modulating regulatory T-cell activity independently from vitamin D production.^31^ However, it is unclear whether the relationship stems from direct exposure in the infant or maternally either during pregnancy or through breastfeeding. A higher risk of infection during the winter could also contribute to the increased MS risk in those born in the fall. This may also explain the trends in the MS risk association as vaccination rates increased after the 1960s resulting in fewer active infections (**Figure 3**). In addition, long-term effects from early and particularly persistent infections (e.g. EBV, HHV6, varicella zoster) may also affect disease progression after MS onset. Our findings show increased disability was also associated with being born in the early fall while younger age at onset was observed for patients born in the summer. Considering that genetic variants in the HLA region are associated with both MS susceptibility and antibody response against infections, certain genetic predispositions might modulate the association between MS and season of birth. However, we observed no difference when stratifying by the carriage of DRB1*15:01 or A*02:01, the most prominent genetic variants associated with MS risk.^32^

Differences in response to infection-related exposures may also explain the stronger risk association among males observed in this study. Passive immunity from breastfeeding is less beneficial for males resulting in a higher risk for neonatal respiratory infections.^33^ Differences in immune response between sexes are also evidenced by the stronger antibody response from early vaccinations for females.^30^ Infections less protected by passive immunity such as the respiratory syncytial virus, rotavirus, and influenza may affect the balance between immune tolerance and resistance during a susceptible period of development, potentially leading to an increased risk of MS. Vitamin D levels may also modulate the severity of the immune responses, which further motivates the possible relationship between the season of birth and MS risk.

In conclusion, our findings suggest that time of birth may influence the risk of MS when factoring in sex, year of birth, and geographical residence; however, the findings will require follow-up and additional validation.

## Abbreviations

ARMSS: Age-related multiple sclerosis severity
EDSS: Expanded disability status scale
EIMS: Epidemiologic investigation in multiple sclerosis
FDR: False discovery rate
GEMS: Genes and environment in multiple sclerosis
HLA: Human leukocyte antigen
IMSE: Immunomodulation and multiple sclerosis epidemiology
MOB: Month of birth
MS: Multiple sclerosis
MSSS: Multiple sclerosis severity score
UVR: Ultraviolet radiation
